# Morphologic criteria of vermiform appendix on computed tomography and a possible risk of developing acute appendicitis

**DOI:** 10.1590/0100-3984.2018.0118

**Published:** 2019

**Authors:** Amanda Chambi Tames, Fernando Ide Yamauchi, Adham do Amaral e Castro, Caroline Duarte de Mello Amoedo, Ellison Fernando Cardoso, Ronaldo Hueb Baroni, Adriano Tachibana

**Affiliations:** 1 Radiology and Diagnostic Imaging, Hospital Israelita Albert Einstein, São Paulo, SP, Brazil.; 2 Instituto de Radiologia do Hospital das Clínicas da Faculdade de Medicina da Universidade de São Paulo (InRad/HC-FMUSP), São Paulo, SP, Brazil.; 3 Department of Diagnostic Imaging, Escola Paulista de Medicina da Universidade Federal de São Paulo (EPM-Unifesp), São Paulo, SP, Brazil.

**Keywords:** Appendicitis, Appendix, Multidetector computed tomography, Emergency medicine, Apendicite, Apêndice, Tomografia computadorizada multidetectores, Medicina de emergência

## Abstract

**Objective:**

To evaluate the correlation of morphological criteria of the cecal appendix using computed tomography (CT) and the possible risk of developing acute appendicitis.

**Materials and Methods:**

Cases were defined as patients with surgically confirmed acute appendicitis who had undergone CT at least twice: at diagnosis and at least one month prior. Controls were defined as emergency patients with abdominal pain who had undergone abdominal CT that excluded acute appendicitis and had also undergone CT at least one month before.

**Results:**

100 cases and 100 controls were selected for inclusion in the final analysis. Comparisons between the cases and controls revealed the following: mean transverse diameter of 0.6 cm (range, 0.4-1.0 cm) versus 0.6 cm (range, 0.6-0.8 cm; *p* = 0.37); mean length of 6.6 cm (range, 3.5-9.7 cm) versus 6.6 cm (range, 4.5-8.3 cm; *p* = 0.87); mean angle of 100° (range, 23-178°) versus 86° (range, 43-160°; *p* = 0.01); vertical descending orientation in 56% versus 45% (*p* = 0.2); absence of gas in 69% versus 77% (*p* = 0.34); and presence of an appendicolith in 17% versus 8% (*p* = 0.08).

**Conclusion:**

Hypothetical risk factors for obstruction of the vermiform appendix detected on CT were not associated with acute appendicitis. That suggests that factors other than those related to mechanical obstruction are implicated in the pathogenesis of acute appendicitis.

## Abstract (pt)

**Objetivo:**

Avaliar a correlação de critérios morfológicos do apêndice cecal por tomografia computadorizada (TC) e o risco de apendicite aguda.

**Materiais e Métodos:**

Casos foram definidos como apendicite aguda confirmada cirurgicamente que tiveram pelo menos dois exames de TC: um no diagnóstico de apendicite aguda e outro no mínimo um mês antes. O grupo controle foi definido como pacientes emergenciais com dor abdominal com TC de abdome excluindo apendicite aguda e com TC prévia pelo menos um mês antes.

**Resultados:**

100 casos e 100 controles foram selecionados. A comparação das variáveis dos casos e controles revelou: diâmetro transverso médio de 0,6 cm (faixa: 0,4-1,0 cm) versus 0,6 (faixa: 0,6-0,8 cm) (*p* = 0,37); comprimento médio de 6,6 cm (faixa: 3,5-9,7 cm) versus 6,6 cm (faixa: 4,5-8,3 cm) (*p* = 0,87); ângulo médio de 100° (faixa: 23-178°) versus 86° (faixa: 43-160°) (*p* = 0,01); orientação descendente em 56% versus 45% (*p* = 0,2); ausência de gás em 69% versus 77% (*p* = 0,34) e presença de apendicólito em 17% versus 8% (*p* = 0,08).

**Conclusão:**

Fatores obstrutivos hipotéticos do apêndice cecal na TC não foram associados a apendicite aguda. Isso sugere que outros fatores diferentes de obstrução mecânica podem estar implicados na gênese da apendicite aguda.

## INTRODUCTION

Acute appendicitis is one of the most common causes of acute abdominal pain and the most common surgical procedure in the emergency department, in adult and pediatric populations, annually accounting for approximately 190,000 hospitalizations in adults^(^^1^^**,**^^2^^)^. The diagnosis of acute appendicitis traditionally relies on history, physical examination, and laboratory tests. Although not mandatory, imaging examinations are very helpful in confirming or excluding the diagnosis in an emergency setting. Regardless of the imaging modality-ultrasound, computed tomography (CT) or magnetic resonance imaging-the findings are quite similar and reflect the chronology of the known physiopathology, including luminal obstruction, appendicular distention, and inflammation, as well as the progression to suppurated transmural inflammation, ischemia, infarction, and perforation^(^^3^^)^.

Given the widespread use of imaging examinations, specifically CT, various incidental findings are being detected in patients. A recent systematic review estimated the frequency of incidental findings on abdominal CT at approximately 30%^(^^4^^)^. In this context, hypothetical predisposing factors, such as an appendicolith and indirect signs of luminal obstruction or near obstruction (for example, the absence of gas inside the appendix) could be observed as incidental findings, potentially increasing the risk of acute appendicitis^(^^5^^)^.

There have been several studies investigating clinical and laboratory findings in patients with acute appendicitis and the risk of developing complications^(^^6^^**,**^^7^^)^, as well as CT findings that can aid in making the diagnosis and predicting complications^(^^8^^**,**^^9^^)^. However, to our knowledge, there have been no studies investigating CT findings as potential risk factors for developing acute appendicitis. Therefore, our objective was to evaluate potential risk factors for the development of acute appendicitis, as detected on CT examinations performed prior to the acute event.

## MATERIALS AND METHODS

This retrospective case-control study was approved by the institutional review board and research ethics committee of our institution. The requirement for individual informed consent was waived. Cases were defined as patients with surgically confirmed acute appendicitis, between January 2009 and December 2016, who had undergone CT at least twice: at diagnosis and at least one month before the acute event. Controls were defined as patients who presented to the emergency department with abdominal pain, during the same period, and had undergone abdominal CT that excluded acute appendicitis, as well as having undergone CT at least one month before the acute event. Cases and controls were matched by gender, age, and body mass index (BMI).

The inclusion criteria were being 18 years of age or older and having undergone abdominal CT in the emergency department. Cases included patients with a radiological, surgical, and histopathological diagnosis of acute appendicitis, whereas controls included those who were discharged from the emergency department. We excluded patients in whom the CT examinations contained artifacts causing image degradation that could impede the analysis.

**Figure 1:**
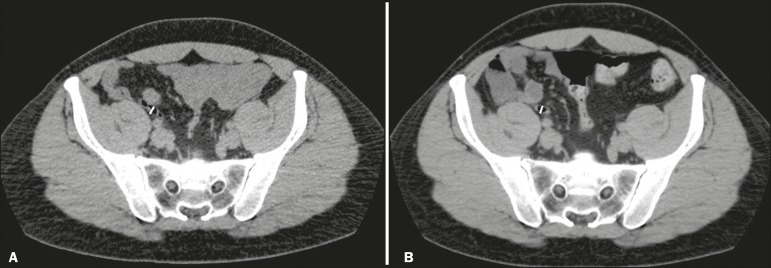
**A:** Transverse diameter of the appendix at its thickest point in the axial plane (6.2 mm). **B:** The same measurement, obtained three years later, after no appendicitis had developed (6.4 mm). Gas within the appendix can be seen in both images.

**Figure 2:**
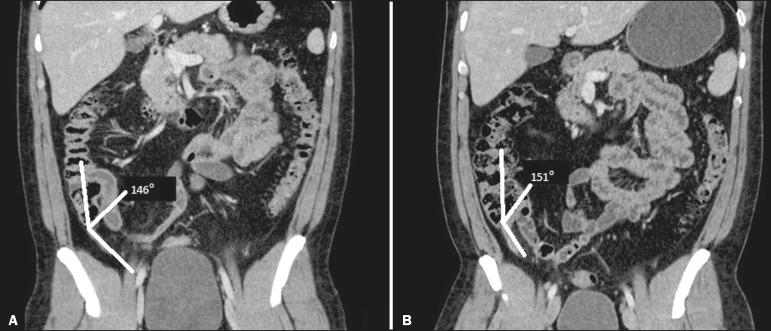
Angle between the cecum and the appendix base. **A:** Normal appendix. **B:** The same patient scanned during the acute event, showing minimal deviation in the angle.

**Figure 3:**
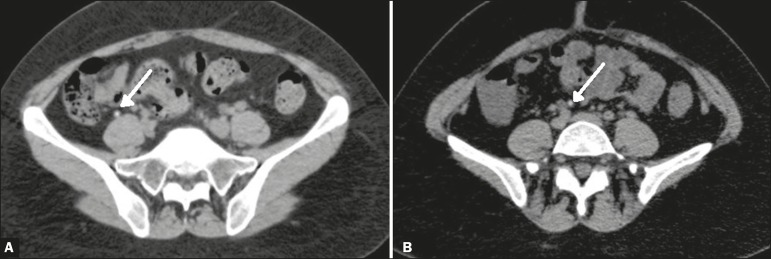
Identification of an appendicolith. **A:** Normal appendix with an appendicolith (arrow). **B:** Five years later, the same appendicolith was identified but no appendicitis had developed.

The following morphological criteria of the cecal vermiform appendix were analyzed on the CT scan acquired prior to the acute event: transverse diameter, length, angle of origin in relation to the cecum, orientation, presence of gas, and presence of an appendicolith. The transverse diameter was measured at the thickest point in the axial plane (Figure 1). The length of the appendix was measured using multiplanar reconstruction tools. The angle was measured on a reconstructed image in the coronal plane; specifically, a vertically descending appendix was considered as a 180° angle between the cecum and the base of the appendix (Figure 2). To evaluate the mobility of the appendix, the angle was measured on both CT scans. A > 90° variation was considered significant. The orientation was classified as vertical ascending, vertical descending, or horizontal, depending on the position of the tip of the appendix. Gas and an appendicolith within the appendix were evaluated as dichotomous variables (present or absent). An appendicolith was defined as a nodular image inside the appendix with attenuation > 150 Hounsfield units (Figure 3).

CT examinations were performed in several different multislice scanners with 16-320 detector-rows (General Electric, Toshiba and Siemens), using a collimation of 2 mm or less, 2.0-mm reconstruction or less, and including an unenhanced phase or post-contrast venous phase (70 sec. delay). Iodinated intravenous contrast media was delivered by power injector at a dose of 1-2 mL/kg of body weight and a rate of 2-3 mL/s.

Statistical analyses were performed using RStudio, version 1.0.153 (RStudio, Inc, Boston, MA, USA). We used a retrograde elimination of variables in a logistic regression model to evaluate association with the future diagnosis of appendicitis and odds ratio of categorical variables.

## RESULTS

An initial search retrieved 2044 cases of acute appendicitis diagnosed on CT during the study period, 523 of the patients having undergone a previous CT examination (at least one month before the acute event). The 100 most recent cases were selected for analysis, and 100 controls (matched by gender, age, and body mass index) were also selected (Table 1). The final cohort for analysis was composed of 200 patients.

**Table 1:** Demographic characteristics of the cases and controls.

Characteristic	Cases (n = 100)	Controls (n = 100)	*P*
Gender			
Male, n	59	57	0.88
Female, n	41	43	
Age (years), mean (range)	43 (18-76)	43 (18-76)	0.98
BMI (kg/m^2^), mean (range)	25.7 (20-39)	26.7 (16-43)	0.10

The mean interval between CT examinations was 39 months (range, 1-108 months) for cases and 41 months (range, 2-112 month) for controls. Overall (cases and controls), the most common orientation was vertical descending (in 50%), followed by vertical ascending (in 35%) and horizontal (in 15%); the mean transverse diameter was 0.6 cm (0.4-1.0 cm); and the mean length was 6.6 cm (3.5-9.7 cm). Significant (> 90°) variations in the angle occurred in 12 (6%) of the patients. Comparing the variables between the cases and controls, using the logistic regression model, we found the data described in Table 2.

**Table 2:** Morphological criteria for CT evaluation of the appendix in cases (patients that later developed acute appendicitis) and controls (patients matched to the cases by gender, age, and BMI).

Criterion	Cases (n = 100)	Controls (n = 100)	*P*
Transverse diameter (cm), mean (range)	0.6 (0.4-1.0)	0.6 (0.6-0.8)	0.37
Length (cm), mean (range)	6.6 (3.5-9.7)	6.6 (4.5-8.3)	0.87
Angle (º), mean (range)	100 (23-178)	86 (43-160)	0.01
Vertical descending orientation, n	56	45	0.20
Absence of gas, n	69	77	0.34
Presence of an appendicolith, n	17	8	0.08

## DISCUSSION

Imaging examinations have become increasingly important in the evaluation of abdominal emergencies^(^^10^^**-**^^12^^)^. Appendicitis is the most common surgical emergency worldwide. Physical examination and laboratory tests, when performed in conjunction with imaging examinations, are highly specific for the diagnosis^(^^13^^)^. It is widely accepted that the pathogenesis of appendicitis involves luminal obstruction followed by distension and inflammation of the appendix^(^^14^^**,**^^15^^)^.

Interestingly, some of the imaging criteria used for the diagnosis of appendicitis on imaging examinations, such as a large transverse diameter, the presence of an appendicolith, and the absence of gas within the appendix, can be seen on CT examinations performed for reasons unrelated to acute appendicitis^(^^14^^**,**^^15^^)^. In our sample as a whole, the baseline CT showed an appendicolith in 12.5% of the patients and the absence of gas within the appendix in 22.5%. Nevertheless, a diagnosis of acute appendicitis was ruled out in all of those patients. In addition, we found a wide range of transverse diameters among the controls. For instance, if a cutoff of 6 mm, a well-established imaging criterion, were applied, 37% of the controls could have been erroneously classified as having acute appendicitis by that single criterion. Similarly, we attempted to determine whether longer appendices with narrower ostia (as indicated by the angle between the appendix base and the cecum) could be at an increased risk for acute appendicitis, as has previously been suggested^(^^16^^**-**^^19^^)^. However, in our study sample, appendix length was comparable between the two groups and a more angled appendix was more prevalent in the control group rather than in the acute appendicitis group. It is noteworthy that there was significant intraindividual variation in the angle in only 12 (6%) of the patients, indicating that the appendix might be more fixed than previously thought.

The morphological aspects of the vermiform appendix seen on CT that could be related to the future development of acute appendicitis, as we hypothesized, are necessarily mechanical factors. However, the modern understanding of the pathogenesis of acute appendicitis suggests that mechanical factors leading to direct lumen obstructions are likely exceptions. The most recent theories regarding the pathogenesis of acute appendicitis involve complex genetic and environmental factors^(^^19^^**-**^^21^^)^. To our knowledge, our study is the first to look for mechanical factors that could be associated with acute appendicitis in an accessible way (via abdominal CT). Although a more in-depth analysis of the pathogenesis of acute appendicitis is beyond the scope of the present study, the absence of statistical associations suggests that exclusively mechanical causes are less important risk factors for the development of acute appendicitis. This underscores the understanding that the causes of and various risk factors for the disease are quite complex, and abdominal CT is unable to provide sufficient information regarding its pathogenesis.

Our study has some limitations. First, the case-control design does not allow us to infer causality. Second, we selected only patients who had undergone at least two CT examinations, which could have introduced a selection bias. Third, the sample size might have been too small to demonstrate statistically significant differences between the groups.

## CONCLUSION

In conclusion, hypothetical risk factors for obstruction of the vermiform appendix detected on CT were not associated with a higher risk of acute appendicitis. The presence of an appendicolith showed only a trend toward an association with appendicitis. Those findings suggest that factors other than those related to mechanical obstruction are implicated in the pathogenesis of acute appendicitis.
